# An optimized stacking-based TinyML model for attack detection in IoT networks

**DOI:** 10.1371/journal.pone.0329227

**Published:** 2025-08-01

**Authors:** Anshika Sharma, Shalli Rani, Mohammad Shabaz

**Affiliations:** 1 Chitkara University Institute of Engineering and Technology, Chitkara University, Rajpura, Punjab, India; 2 Marwadi University Research Center, Department of Computer Engineering, Faculty of Engineering and Technology, Marwadi University, Rajkot, Gujarat, India; Cardiff Metropolitan University - Llandaff Campus: Cardiff Metropolitan University, UNITED KINGDOM OF GREAT BRITAIN AND NORTHERN IRELAND

## Abstract

With the expansion of Internet of Things (IoT) devices, security is an important issue as attacks are constantly gaining more complex. Traditional attack detection methods in IoT systems have difficulty being able to process real-time and access limitations. To address these challenges, a stacking-based Tiny Machine Learning (TinyML) models has been proposed for attack detection in IoT networks. This ensures detection efficiently and without additional computational overhead. The experiments have been conducted using the publicly available ToN-IoT dataset, comprising a total of 461,008 labeled instances with 10 types of attacks categories. Some amount of data preprocessing has been done applying methods such as label encoding, feature selection, and data standardization. A stacking ensemble learning technique uses multiple models combining lightweight Decision Tree (DT) and small Neural Network (NN) to aggregate power of the system and generalize. The performance of the model is evaluated by accuracy, precision, recall, F1-score, specificity, and false positive rate (FPR). Experimental results demonstrate that the stacked TinyML model is superior to traditional ML methods in terms of efficiency and detection performance, and its accuracy rate is 99.98%. It has an average inference latency of 0.12 ms and an estimated power consumption of 0.01 mW.

## 1 Introduction

The proliferation of IoT has revolutionized industries by means of an efficient automation and communication support. IoT networks are simple prey to attackers due to the presence of devastating vulnerabilities generated by its growth [1]. These comprise botnet attacks, DDoS attacks, malware-infections, which may be very risky to the IoT environment and put at risk user data [2]. Therefore, there is an increasing need for advanced threat detection systems that are portable and efficient, which provides a motivation for studying on [3].

Existing Studies show that ML based IDS are promising for detecting cyber threats online [4,5]. But conventional ML models are not applied to limited resource IoT devices due to its high processing overhead [6]. Such are the main problems that TinyML addresses, which allow ML models to directly run on edge devices at real-time low power consumption, is one of the plausible solutions to these problems [7]. TinyML-powered IDS are able to overcome latency and confidentiality concerns, offering enhanced security by eliminating the reliance on cloud-based technologies [8]. Apart from its compatibility with the IoT, there are other advantages offered by TinyML that are especially helpful within practical security contexts. One of its great attributes is its extremely low power consumption, so it works well on batter and is long-lasting, needing to be replaced only after long intervals. IoT sensors located in remote or inaccessible settings need this capability. Furthermore, TinyML allows for real-time inference, which is important for reducing latency by not having to send data to central cloud servers for processing [9]. It not only enhances the speed, but also makes the security and privacy of data better because the private data could be computed and analyzed locally and be processed without leaking network flaws. Small and lightweight, TinyML models can be deployed on embedded devices and micro-controllers with limited memory and processing resources [10]. Added together the combination makes TinyML an inexpensive, secure and anonymous method for disseminating low cost and reliable intrusion detection systems in the emerging IoT landscape [11]. In this work, a Stacking TinyML model has been proposed to address these challenges and train and test an attack detection procedure using the ToN IoT dataset [12].

In this work, the ensemble approach is used to exploit the prediction ability of many base models to enhance overall performance. First, the process is initialized with a few basic learners that are learned independently on the same dataset ToN-IoT. Next, a meta-classifier is fed with their predicted outputs as input features and trained to optimally combine them to output the final predictions. Here the strengths of two models including lightweight DTs and small NNs has been stacked, both TinyML-optimal to an extent. Generalization, detection accuracy, and dependability are enhanced by meta-classifying the actual classifier using a Logistic Regression (LR) on the results of the base learners. The model has also been compared with the existing work including Kalidindi *et al*. [13], Arcot *et al*. [10], Agrawal *et al*. [9], Alwaisi *et al*. [14], Odat *et al*. [2] and Katib *et al*. [4] used the different ML and TinyML techniques achieving the accuracies of 92.4%, 94.90%, 96.45%, 96.9%, 98% and 98.11% respectively. By utilizing an optimized meta-classifier LR and combining the individual strengths of multiple base learners, the model is able to overcome the limitations of individual TinyML models. Moreover, the ToN-IoT dataset comprised of real IoT traffic data provides a comprehensive testbed for measuring model performance under a variety of attack conditions.

### 1.1 Contribution

The main contribution of the paper is as follows:

- Proposed a TinyML model for attack detection based on Stacking that improves detection accuracy in IoT networks by introducing an ensemble strategy combining small NN and lightweight DT.
- Adapted to IoT devices with limited resources guarantees extremely low latency (0.12 ms) and low power consumption (0.01 mW), which qualifies it for microcontroller real-time deployment.
- Utilized feature selection techniques to improve classification performance, the most pertinent features are chosen using the small random forest (RF).
- Outperforms traditional ML models and single TinyML in attack detection, achieving 99.98% accuracy using the ToN-IoT dataset.
- Highlights the stacking method’s resilience, scalability, and efficiency in identifying changing cyber risks, proving its superiority over traditional TinyML models.

### 1.2 Structure of the paper

The structure of the paper is organized as follows: The Introduction section explains about the motivation, problem description, and contribution. The Literature review section reviews the attack detection in IoT networks utilizing TinyML and conventional ML approaches. The proposed methodology section proposes the architecture of the lightweight DT, the small NN, and the stacking-based TinyML model along with the dataset description and data preprocessing techniques. The Results and analysis Section describes the experimental results which are examined thoroughly and covers performance indicators including accuracy, precision, recall, F1-score and inference latency. Conclusion and future work Section concludes the study and suggests possible work for future research.

## 2 Literature review

An ML-based approach to Android malware detection that utilizes co-existing static features such as permissions and API calls is introduced by Odat *et al*. [2]. The model supposes that, unlike benign applications, malware needs unusual APIs and permissions. In order to verify this, Drebin, Malgenome, and MalDroid2020 Android APK datasets were used to generate a dataset with co-existent features at varied combination levels. Various ML classifiers were used once the most prominent features were acquired with the help of the FP-growth approach. Using the RF classifier, model accuracy was maximum at 98%. By reaching 98% accuracy for Malgenome and 95% for Drebin, against 87% and 93%, respectively. Botnet attacks pose a significant cybersecurity threat as they allow hackers to exploit connected devices for harmful purposes. Kalidindi *et al*. [13] suggested a Deep Learning (DL) botnet detection model for IoT networks. The procedure starts with the collection of log data and then quantile normalization for data preparation. Feature selection is performed using Information Gain (IG) and City Block Distance, and oversampling is used for data augmentation. A Convolutional Neural Network Integrated with Deep Stacked Autoencoder (CNN-FDSA) is employed to output the final detection. The model presented achieves efficient botnet detection with minimal False Positive Rate (8.2%) while achieving higher accuracy (92.4%), recall (90.3%), and precision (91.6%).

Dai *et al*. [15] uses autoencoders for anomaly detection using the CIC-MalMem-2022 dataset to address the difficulty of current intrusion detection systems in detecting zero-day attacks. After integrating the trained autoencoder with RF and XGBoost, the models XGBoost-AE and RF-AE are produced. Using TensorFlow Lite Micro and lightweight ML models on microcontrollers, Agrawal *et al*. [9] created TinyAP, an intelligent access point. A Raspberry Pi 3 B+ serves as the system’s access point, while an Arduino Nano 33 BLE Sense runs Pretrained TinyML models. Multilayer Perceptron (MLP) models were optimized for deployment using Neural Architecture Search (NAS). TinyAP demonstrated 96.45% average accuracy in testing on a dataset of 13 different forms of Wi-Fi attacks, Comparing multiclass classification to traditional MLP models, the accuracy was 95.19% with just slight decreases.

IoT environments rely on edge devices, but due to their low storage and processing capacities, they are susceptible to security threats. Latency caused by conventional ML models running on GPUs, CPUs, and cloud servers delays the detection of threats. To circumvent this, Arcot *et al*. [10] employ TinyML to model optimization for resource-constrained edge devices via TensorFlow Lite. Their tests on the USTC-TFC2016 and Ransomware PCAP datasets examine the quantization technique (QAT) by striking a balance between inference time, model size, and accuracy. QAT achieved 94.90% accuracy on the USTC-TFC2016, and static int8 reduced the model size to 12.46 MB. By lowering DenseNet169’s size to 23.99 MB while maintaining 38.89% accuracy for the Ransomware dataset, static float16 illustrated TinyML’s trade-offs for apps related to cybersecurity. Mohamad *et al*. [16] suggests evaluating Android malware detection using several ML approaches including RF, AdaBoost, J48, MLP and KNN. To find out which traits were best in differentiating malware, this study used the feature selection approach. Five thousand samples of Drebin malware and 5,000 samples of Androzoo benign samples were used in total.

Oliullah *et al*. [17] employ a two-step authentication procedure, which begins with anonymous authentication utilizing a secret ID using Elliptic Curve Cryptography (ECC). For users whose behavior is deemed questionable, an intrusion detection technique is subsequently used. A suitable method for the integration of ML-based attack detection within IoT systems is tinyML. Alwaisi *et al*. [18] compared ML models and their consumption of power, memory, and efficiency for detecting attacks in which limited resources are analyzed. Based on their findings, the DT model itself is advantageous than other options. The possibility of the TinyML model for IoT network security is proven using 96.9% accuracy. This work paves the way for realizing TinyML based security solutions in real-time IoT systems.

Arshad *et al*. [19] proposed a Trust-based Hybrid Cooperative RPL protocol (THC-RPL) that identifies malicious Sybil nodes in an RPL-based IoT network. Since the requirements of the IoT are very different from what the Internet can provide at the moment, the IoT has increasingly incorporated disparate technologies like Wireless Sensor Networks (WSN) and Mobile Ad hoc Networks (MANETs). Using Z-score normalization for the feature extraction by Fennec Fox Optimization Algorithm (FFA) and the GLMS-BiLSTM for anomaly detection, Katib *et al*. [4] present the DLTML-RTADPM (TinyML Driven Real-time Anomaly Detection for Predictive Maintenance) technique. The JOA is applied for hyperparameters tuning of detection performance. In as much as anomaly detection in IoT environments is concerned, experimental results show that the DLTML-RTADPM performs better than other methods with a remarkable accuracy of 98.11%. Towards enhancing malware detection efficacy and precision, Kauser *et al*. [20] provides a hybrid deep learning model (DBN-GRU) that integrates Gated Recurrent Units (GRU) for dynamic behavior modeling and Deep Belief Networks (DBN) for static analysis. Precise analysis of application behavior is facilitated by the model’s extraction of static (permissions, API calls, intent filters) and dynamic properties from Android APKs.

Yang *et al*. [21] proposes a cloud-edge collaborating data anomaly identification technique for industrial sensor networks using sensor data detection models at edges and cloud-based analysis models. The former uses Gaussian and Bayesian methods to filter the large amount of data from sensors generated during industrial sensor network operation, lowering traffic load. Only when the network is abnormal does it upload every sensor’s information to the sensors data evaluation model for analysis. GCRL-based anomaly detection uses Long Short-Term Memory network (LSTM) and Graph Convolutional Network (GCN) to extract spatial and temporal sensor data features. The suggested method is extensively tested using two open industrial sensor network datasets and baseline anomaly detection models. Tawfik *et al*. [22] suggests a unique framework for intrusion detection in fog and IoT networks that combines stacked autoencoders, CatBoost, and an optimized transformer-CNN-LSTM ensemble. Autoencoders reduce the dimensionality of the efficiency at fog nodes while extracting robust characteristics from high-dimensional traffic data. CatBoost uses predictive selection to hone characteristics. For thorough cloud traffic analysis, the ensemble model integrates recurrence, convolutions, and self-attention. Sankaranarayanan *et al*. [23] presented a stacking-based ensemble classifier to multi-class classify harmful URLs on larger URL datasets to demonstrate its robustness. This study employs URL lexical properties to identify fraudulent websites. RF, XGBoost, LightGBM, and CatBoost are used to create the stacking-based ensemble classifier. Additionally, the proposed classifier was optimized using Randomized Search hyperparameter tuning. The stacking-based ensemble classifier aggregates ML model output to improve prediction accuracy. RF, XGBoost, LightGBM, and CatBoost have classification accuracies of 93.6%, 95.2%, 95.7%, and 94.8%, respectively. The stacking-based ensemble classifier can classify four dangerous URL classes (phishing, malware, defacement, and benign) with 96.8% accuracy.

### 2.1 Summary

ML and DL techniques for identifying malware, botnet attacks, and network intrusions have been thoroughly studied in recent IoT and mobile security research. While some studies have relied on network traffic data and system logs, others have used static elements from Android APKs, such as permissions, intent filters, and API calls. To improve classifier performance, feature selection techniques such as FP-Growth, Information Gain, and FFA have been used. Models with high detection accuracy often over 90% include several ML and DL techniques like RF, CNN-Autoencoders, DBN-GRU hybrids, and stacking-based ensembles. The efficacy of these methods has been demonstrated by validation on popular datasets such as Drebin, BoT-IoT, CIC-MalMem-2022, and Ransomware PCAP. However, the actual implementation of these models in edge-based IoT networks is limited due to their need on huge memory, centralized processing, and high computational power.

TinyML has surfaced as a promising substitute to overcome these constraints, allowing ML inference on microcontrollers and ultra-low-power devices. Unlike GPU- or cloud-based ML and DL models, tinyML models use pruning, quantization, and structural search to reduce size, memory, and energy usage. This makes them perfect for real-time attack detection on small devices like the Raspberry Pi Pico, Arduino Nano, and ESP32. Deploy models at the edge to reduce latency and eliminate the need for continuous internet access, which are crucial for mission-critical IoT applications. Research like TinyAP and DLTML-RTADPM shows that TinyML-based approaches are more efficient, responsive, and accurate than other methods, making them the best choice for next-generation IoT network security.

## 3 Proposed methodology

**Fig 1:**
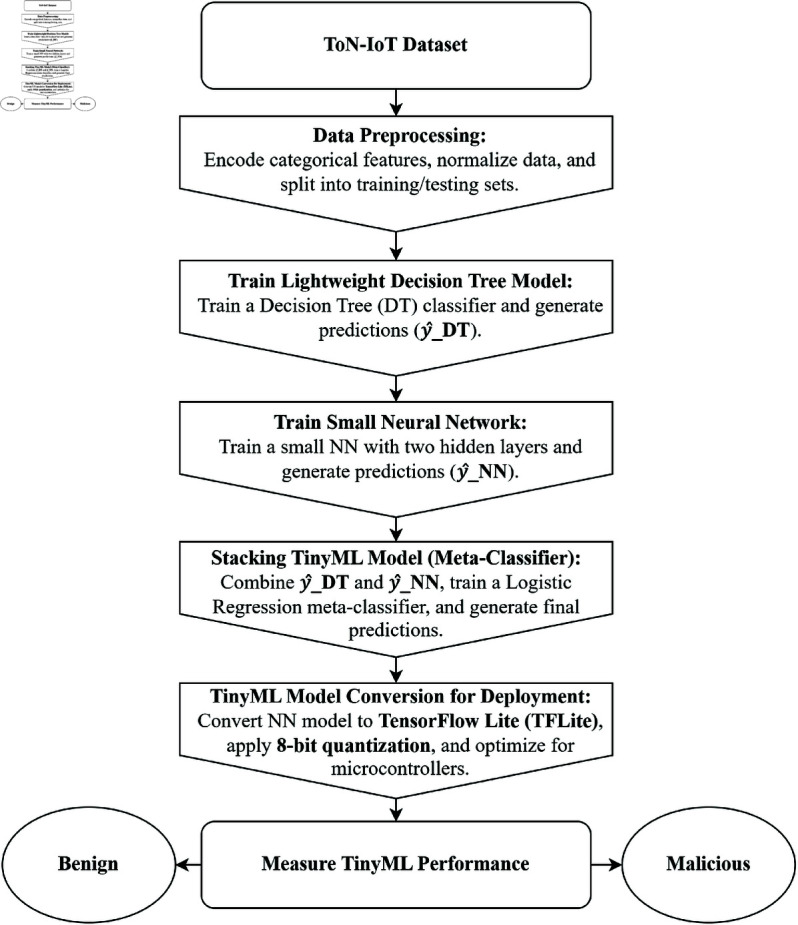
Proposed methodology.

This section represents the proposed methodology developing a stacking-based lightweight TinyML model combining the strengths of lightweight DL and small NN models for the identification of attacks in IoT networks. The methodology includes several steps like dataset overview, data preparation, model training and the proposed TinyML model architecture, also shown by the Algorithm 1. Utilizing quantization and other optimization methods, the model has been optimized for low-power inference, enabling real-time microcontroller deployment. A detailed explanation of each step is provided in Fig 1.

### 3.1 Dataset description

Network traffic from IoT networks and data from IoT services developed by UNSW Canberra’s Cyber Range and IoT Labs are among the many sources of data included in the TON-IoT dataset collection at https://research.unsw.edu.au/projects/toniot-datasets [24]. They stored the generated data in a CSV file. The two main directories for the provided datasets are “Processed datasets” and “Train Test datasets.” In the “Processed datasets” subdirectory, CSV files containing a prepared version of the datasets with their typical attributes and labels are kept. Sample datasets from the ‘Train Test datasets’ folder are used as train-test datasets in a CSV format that are selected to assess the accuracy and efficacy of ML and cybersecurity technologies. There are a total of 461,043 samples with 45 features in the dataset. A ‘label’ feature distinguishes between malicious and usual data and a ‘type’ feature distinguishes between the various attack types including ransomware, XSS, backdoors, injections, DoS, DDoS, passwords, scanning, and MITM are built into the suggested dataset. Table 1 represents the attack statistics of different classes.

**Table 1:** Attack statistics of different classes.

Class	Attack	Value
0	Backdoor	20,000
1	DDoS	20,000
2	DoS	20,000
3	Injection	20,000
4	MITM	1043
5	Normal	300,000
6	Password	20,000
7	Ransomware	20,000
8	Scanning	20,000
9	XSS	20,000

### 3.2 Data preprocessing

To ensure increased accuracy and efficiency, data preparation is an essential step in getting raw data ready for ML models [25]. Categorical feature encoding, feature selection, and dataset splitting into training and testing sets are some of the sub-steps involved. These procedures aid in converting unstructured data into a format that can be used for evaluation and modeling, especially in TinyML contexts where computational performance is crucial [26].

#### 3.2.1 Encoding techniques.

Categorical variables included in real-world datasets frequently need to be transformed into numerical form to be utilized in ML models. Encoding is the term for this conversion procedure where Label Encoding, which gives categories numerical values has been used in this study. To improve memory efficiency, especially in TinyML contexts with limited resources, Label Encoding was selected over One-Hot Encoding. For embedded devices, One-Hot Encoding is not feasible because to its substantial increase in dimensionality, which raises memory consumption and computational overhead. To ensure effective model execution without requiring excessive resource demands, Label Encoding preserves categorical linkages while maintaining a compact representation [14].

#### 3.2.2 Feature selection.

To increase model efficiency, feature selection entails determining which features are most pertinent while eliminating superfluous, redundant, or highly linked features. Choosing lightweight features is crucial for TinyML applications to save memory and computational effort. Table 2 represents the top 15 selected features from the dataset using the small RF. The reduction improves the efficiency of ML models in dimensionality, which qualifies them for use on microcontrollers with constrained processing capability [27]. A lightweight RF has fewer trees and less depth than typical RF models, which can be challenging to compute. This makes it appropriate for contexts with limited resources, like microcontrollers. By ranking features according to their relevance ratings, this strategy eliminates redundant or unimportant attributes while enabling the selection of the most significant ones. The model achieves faster inference, reduced memory consumption, and enhanced generalization by shrinking the feature space all of which are critical for TinyML implementation. RF is a great option for choosing features that optimize classification performance while lowering computing overhead because it is also resistant to noise and capable of handling non-linear relationships. To differentiate between malicious and legitimate network activity, the selected features are essential.

**Table 2:** Selected features of the ToN-IoT dataset.

Features	Importance	Description
type	0.228743	Type of network flow.
srcip	0.206523	Source IP address of the network packet.
ts	0.102907	Timestamp of the network packet capture.
proto	0.085659	Protocol used in the packet.
dstport	0.071572	Destination port number associated with the packet.
dstip	0.057167	Destination IP address of the network packet.
srcipbytes	0.049785	Number of bytes transmitted by the source IP.
srcpkts	0.031956	Number of packets sent to the source IP.
dstippkts	0.0266630	Number of packets sent to the destination IP.
connstate	0.022293	Connection state.
srcport	0.019904	Source port number used for communication.
dstpkts	0.019423	Number of packets received by the destination IP.
duration	0.012966	Duration of the connection in seconds.
dstbytes	0.017122	Number of bytes received by the destination IP.
dnsRD	0.010310	DNS Recursion Desired flag, indicating if recursive query resolution is requested.

#### 3.2.3 Data standardization and class imbalance.

Data Standardization helps TinyML models achieve better latency inference efficiency on microcontrollers, increasing convergence, and reduce power consumption [28]. There is also a class imbalance where, 161,043 are the attack occurrences against 300,000 normal samples. The Synthetic Minority Over-sampling Technique (SMOTE) has been used to create synthetic samples for the minority class to rectify this imbalance. This technique balances the dataset by learning the model meaningful patterns for all threat types, improving detection accuracy and reducing majority class biasness.

#### 3.2.4 Dataset splitting.

The preprocessed dataset has been split into training (80%) and testing sets (20%) to see how well the model works. The training set is used to fit the models, while the testing set helps to measure how well they generalize.

**Algorithm 1. Stacking TinyML model workflow.**

1: **Input:** ToN-IoT Dataset

2: **Output:** Optimized Stacking TinyML Model for

Microcontrollers

3: **Step 1: Data Preprocessing**

4: Encode categorical features using LabelEncoder

5: Split dataset into features X and labels y

6: Convert y into a multi-class format using label binarization

7: Normalize X using MinMaxScaler (suitable for embedded   devices)

8: Dataset Splitting into training and testing sets (80:20   respectively)

9: **Step 2: Train Lightweight Decision Tree Model**

10: Define a lightweight DT with OneVsRestClassifier(DecisionTreeClassifier

(max_depth=5, min_samples_split=5))

11: Train DT on $X_{train},y_{train}$

12: Predict ${\hat{y}}_{DT}\leftarrow DT.predict(X_{test})$

13: **Step 3: Train Small Neural Network**

14: Define a small NN with only two hidden layers ([32, 16])

15: Use ReLU activation and optimize with Adam

16: Train NN on $X_{train},y_{train}$ using 32 batch sizes

17: Predict ${\hat{y}}_{NN}\leftarrow NN.predict(X_{test})$

18: **Step 4: Stacking TinyML Model (Meta-Classifier)**

19: Combine ${\hat{y}}_{DT}$ and ${\hat{y}}_{NN}$ as input to meta-classifier

20: Use lightweight Logistic Regression as a meta-classifier

21: Train the meta-classifier on combined predictions

22: Predict ${\hat{y}}_{stackingTinyML}\leftarrow Meta.predict([{\hat{y}}_{DT},{\hat{y}}_{NN}])$

23: **Step 5: TinyML Model Conversion for Deployment**

24: Convert trained NN model to TensorFlow Lite format using

TFLiteConverter

25: Apply quantisation (8-bit) for memory efficiency

26: Optimize conversion for microcontrollers

27: Save the converted model as stacking

TinyML_tinyml_model.tflite

28: **Step 6: Measure TinyML Performance**

29: Load stacking TinyML_tinyml_model.tflite using TensorFlow

Lite Interpreter

30: Measure inference latency on test samples

31: Estimate power consumption using TinyML benchmarks

32: Compute all the performance metrics of the TinyML models

### 3.3 Model training

A critical component in TinyML is model training, which entails maximizing ML models’ performance on resource-constrained embedded systems. For real-time prediction on controllers and low-power edge devices, TinyML models need to be computationally streamlined, lightweight, and memory-efficient, in contrast to typical ML models made for high-performance computing environments. Feature and model selection, optimization methods, and transformation into a deployable format, such as TensorFlow Lite, are all part of the training process. Since they strike a mix between accuracy and processing efficiency, DT and small NN are the most popular models employed in TinyML. The OneVsRestClassifier has been used with the DT model to handle multi-class classification effectively by breaking it down into multiple binary classification problems. This approach allows the model to independently learn decision boundaries for each class, improving interpretability and computational efficiency in TinyML environments. Additionally, it ensures flexibility in handling imbalanced class distributions within the dataset. Compact datasets are used to train these models, and lightweight activation functions, quantization, and pruning are used to optimize them. The lightweight DT and small NN for TinyML are defined as:

#### 3.3.1 Decision tree for TinyML.

A decision tree is a tree-like framework in which the leaves stand for the anticipated class and each node for a decision rule determined by a feature [29]. Being based on splitting the feature space, it is suitable for TinyML classification tasks which are not too hard. In practice, a DT uses a recursive splitting rule based on the entropy or Gini impurity at each node in the tree. The entropy and the information gain (IG) of a dataset *S* for the splitting criteria are calculated by Eqs 1 and 2.

$$H(S)=-\sum_{i=1}^{c}p_{i}\log_{2}p_{i}$$ (1)

where *p*_*i*_ is the instance proportion belonging to class *i*.

$$IG=H(S)-\sum_{j=1}^{m}\frac{\left|S_{j}\right|}{\left|S\right|}H(S_{j})$$ (2)

Another common criterion for splitting nodes in DTs is the Gini impurity represented by Eq 3:

$$Gini(S)=1-\sum_{i=1}^{c}p_{i}^{2}$$ (3)

Lightweight DT is optimized for TinyML, through the use of quantization to effectively store decision nodes, pruning strategies to eliminate superfluous nodes and avoid overfitting, and depth limitation to minimize memory usage.

#### 3.3.2 Neural network for TinyML.

A neural Network is a computing model made up of several layers of neurons, each of which applies a non-linear activation function after a weighted transformation [30]. The objective of TinyML is to preserve accuracy while keeping the small NN. Each neuron in a layer applies the transformation as given by Eq 4:

z=wx+b (4)

where *w*, *x* and *b* are the weight matrix, input feature vector and the bias term respectively. The output of each neuron is passed through an activation function say the Rectified Linear Unit (ReLU) shown by Eq 5:

a=max(0,z) (5)

A small NN used in TinyML is typically structured as given by Eq 6:

$$f(X)=\sigma (w_{2}\cdot \sigma (w_{1}\cdot x+b_{1})+b_{2})$$ (6)

where σ represents an activation function. TinyML optimizes small NN by using a minimum number of hidden layers, limiting the number of neurons per layer to a minimum of 16 or 8 and selecting an effective activation function.

TinyML models optimize DT and small NN to achieve low latency, minimal power consumption, and efficient inference, which makes them perfect for deployment on edge devices. Table 3 shows the hyperparameters used for the proposed TinyML model.

**Table 3:** Hyperparameters for small NN and lightweight DT.

Model	Hyperparameter
Small NN	Number of Layers: [32, 16]
	Activation Function: ReLU
	Optimizer: Adam
	Learning Rate: 0.001
	Batch Size: 32
	Loss Function: Categorical Cross-Entropy
	Regularization: Dropout (0.2)
Lightweight DT	Splitting Criterion: Gini Index
	Max Depth: 5
	Min Samples Split: 5
	Min Samples Leaf: 1
	Loss Function: Gini Impurity
	Regularization: Pruning

### 3.4 TinyML model architecture

TinyML application framework is designed for real-time inference, memory management and computational optimization on resource constrained embedded devices. In a stacked TinyML with DT and NN, DT can act as a rule-based classifier, and the NN boosts learning ability through the advanced pattern recognition. Stacking TinyML combines the advantages of the two: NN’s capability of learning and DT’s interpretable and low latency properties. DT makes rule-of-thumb decisions for simple cases used as a prefilter, while NN calibrates predictions for uncertain cases. Such a design can also be supported by TinyML applications because it guarantees light-hearted processing and finds a tradeoff between correctness and throughput. A light LR model for the meta classifier has been considered. Low processing time, and ability to be used for TinyML applications led to selecting LR as the meta-classifier. LR effectively integrates the predictions of base learners without adding substantial memory or computation load, which is different from any complicated ensemble approach. Moreover, LR is the best choice for the resource-limited edge devices, since its capability of multi-class classification with low runtime. The three models (DT, NN and LR) were then quantised to TFLite models using TensorFlow Lite Interpreter and run on the same microcontroller.

#### 3.4.1 Transformation a neural network into TFLite for TinyML.

To use an NN on TinyML platform, it must be converted to a TFLite-model, which is optimized for an embedded system. It begins with converting a trained NN in TensorFlow to the TFLite format. Then, notes are serialized and the corresponding metadata is removed from the model and the storage is compacted. Then, at the second stage, the floating-point weights are quantized to a low level of precision, which consumes significantly less memory, but do not lose precision. The model is post-trained after quantization optimizations, such as operator fusion and pruning, to further shrink down the network size while preserving the important characteristics. The converted TFLite model is executed on an microcontroller using a performance-optimized TFLite interpreter, which in turn can perform inferences efficiently with hardware acceleration provided it is supported.

#### 3.4.2 Optimization methods for inference.

This study uses 8-bit post-training quantization to change the weights of models and stimulation into less accurate integers after training. This makes it possible to do integer-based computations with the least amount of memory and inference delay. Pruning is also used to get rid of unnecessary connections in the NN, which makes it easier to work with. By combining several operations into one computing step and therefore reducing overhead, operator fusion improves efficiency even more. By using the knowledge of a more extensive, more complicated model, shared weighting and knowledge distillation enable a smaller, renewable model to preserve predictive performance. The TinyML model is fit for embedded AI and IoT applications since these optimization methods provide real-time inference with low power consumption.

### 3.5 Model evaluation

The stacking TinyML model’s performance is evaluated in TinyML’s final model evaluation, taking into account both embedded device limitations and traditional ML metrics. First, using actual test data, the stacking TinyML model’s accuracy, precision, recall, specificity, FPR and F1-score are calculated. Since efficiency is the main goal of TinyML, other metrics like memory, power consumption, and inference delay are also measured. TFLite, a version of the NN component, is tested on an embedded device to see how fast and how much power it uses. The DT component ensures low computing overhead by operating under a set of predetermined conditions. To ensure the model satisfies resource-constrained edge devices’ requirements while preserving dependable attack detection performance, these metrics collectively have been analyzed to identify the trade-offs between accuracy and efficiency.

## 4 Results and analysis

In this section, the proposed stacking TinyML model has been evaluated and tested by various performance metrics, such as specificity, accuracy, precision, recall, F1-score and False Positive Rate (FPR).

### 4.1 Experimental set-up

An ARM Cortex-M4 processor with 256KB of RAM powered an Arduino Nano 33 BLE Sense development board, which was used for all inference latency and power measurements. Cortex-M series processor-calibrated TinyML benchmark tools were used to assess power usage. The findings show performance under actual embedded deployment situations. TensorFlow Lite Interpreter for Microcontrollers was used to quantify latency.

The prediction results are evaluated based on the confusion matrix that gives information about the classification performance of the model. To provide a more detailed description about how the stacking model improves the attack detection in IoT environments with limited resources, we also had a look at the comparisons between single TinyML classifiers (the DT and the NN), and the TinyML stacking model. One possible way of visualizing the performance of a classification model is through a confusion matrix, showing the percentage of correct and incorrect predictions made by the various classes. It can be formulated by an N×N matrix, where N denotes the number of classes. Each row is the actual class and each column is the predicted class. Here are some terminologies:

- True Positives (TP): Positive cases that were accurately predicted.
- True Negatives (TN): Negative cases that were accurately predicted.
- False Positives (FP): Type I errors that result in incorrectly predicted positive cases.
- False Negatives (FN): Type II errors that result in incorrectly predicted negative cases.

**Fig 2:**
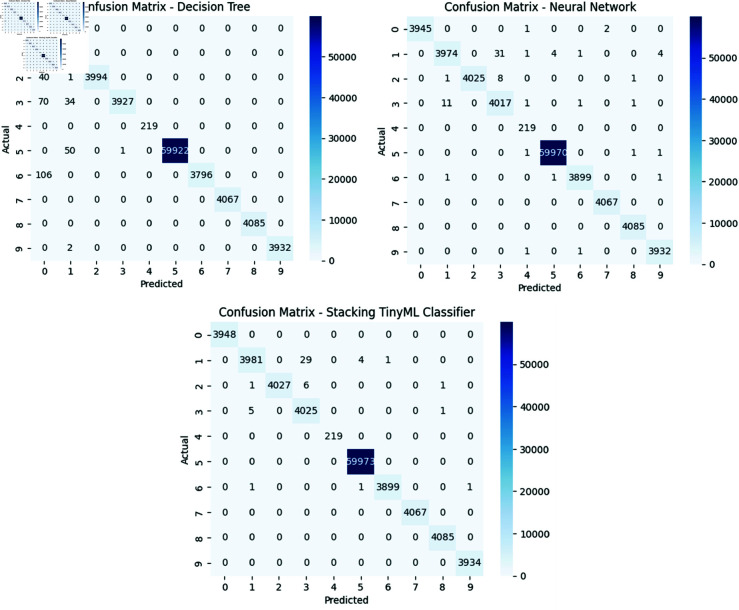
Confusion matrices for the DT, NN and stacking TinyML models.

The confusion matrices of the DT, NN and stacking TinyML models are illustrated in Fig 2. NN model has fewer misclassified samples, so it is more accurate for each class. DT instead has more misclassified samples from Various classes. The ability of stacking TinyML classifier to enhance the accuracy of attack detection is demonstrated in the fact that predictions are more accurate and stable for each class, but with reduced number of errors.

**Fig 3:**
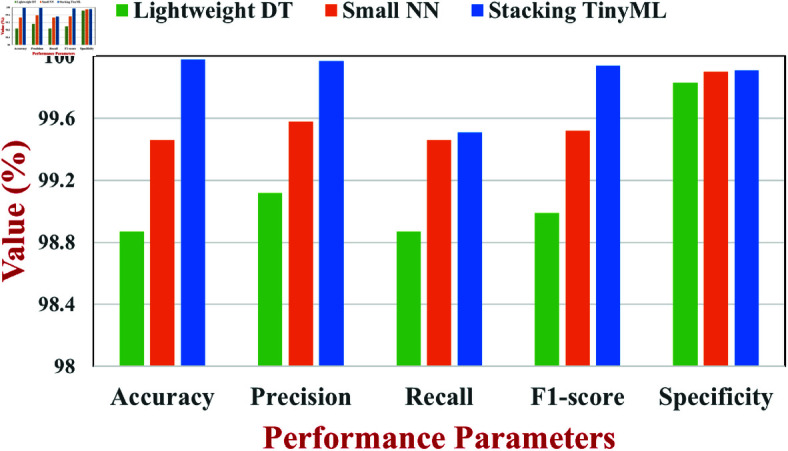
Performance metrics of the TinyML models.

Some performance measurements of three models including our proposed Stacking TinyML, Small NN, and Lightweight DT are compared in Table 4. Some of these measures, such as precision, recall, F1-score, specificity, and PR, are used to offer a comprehensive evaluation of the classification performance of each of the model. It refers to the proportion of the total number of the test samples that were classified correctly by the model. It is one of the most important metrics of overall performance that is utilized for measuring classification models. Fig 3 shows the accuracies of different models as Lightweight DT model’s accuracy is 98.87%, Small NN model’s accuracy is 99.46%, and Stacking TinyML model’s accuracy is 99.98%. A ratio of correctly predicted positive instances to all anticipated positive instances is referred to as precision. A more precise model is more reliable in detecting actual threats since it generates fewer FP. Minimal erroneous positive predictions are indicated by the Lightweight DT model’s 0.9912 precision, the Small NN’s 0.9958 precision, and the Stacking TinyML model’s 0.9997 precision.

**Table 4:** Comparison and analysis of performance metrics using ToN-IoT dataset.

Models	Parameters					
	Accuracy	Precision	Recall	F1-Score	Specificity	FPR
Lightweight DT	0.9887	0.9912	0.9887	0.9899	0.9983	0.0017
Small NN	0.9946	0.9958	0.9946	0.9952	0.9990	0.0010
**Stacking TinyML**	**0.9998**	**0.9997**	**0.9991**	**0.9994**	**0.9991**	**0.0009**

Recall quantifies the percentage of real positive cases that the model accurately detects; it is often referred to as sensitivity or true positive rate (TPR). A high recall value guarantees that the majority of positive cases are effectively captured by the model. Lightweight DT, Small NN, and Stacking TinyML have respective recall values of 0.9887, 0.9946, and 0.9951. The Stacking TinyML model strikes the best compromise between identifying positive cases and lowering FN, even though all three models show high recall scores. The model’s classification performance is balanced by the F1-score, which is the harmonic average of precision and recall. A favourable trade-off between precision and recall is demonstrated by the F1-scores of 0.9899 for the Lightweight DT model, 0.9952 for the Small NN model, and 0.9994 for the Stacking TinyML model. The percentage of accurately detected negative cases is measured by specificity. When the specificity is greater, the normal and attack events are well differentiated from the model. The specificity scores for Lightweight DT, Small NN, and Stacking TinyML model are 0.9983, 0.9990, and 0.9991, respectively. One of the critical statistics involved in cybersecurity applications is the False Positive Rate (FPR) which denotes the percentage of negatives wrongly classified as positive. Since lower FPR means true non-attacks are less likely to be false “positives”, smaller FPR is desired. The ability of the Lightweight DT in avoiding false alarms is reflected by its 0.0017 FPR, while Small NN and Stacking TinyML have 0.0010 and 0.0009 FPR, respectively. The average inference latency of the TinyML models, as well as their classification accuracy, is 0.12 milli-seconds, which ensures that the models can run in real-time on resource-constrained devices. Moreover, the estimated power consumption of the final Stacking TinyML model is 0.01 mW, which is very low to be fitted in energy-constrained edge devices. These characteristics show how good a compromise the Stacking TinyML model is between accuracy and low latency and low power.

**Fig 4:**
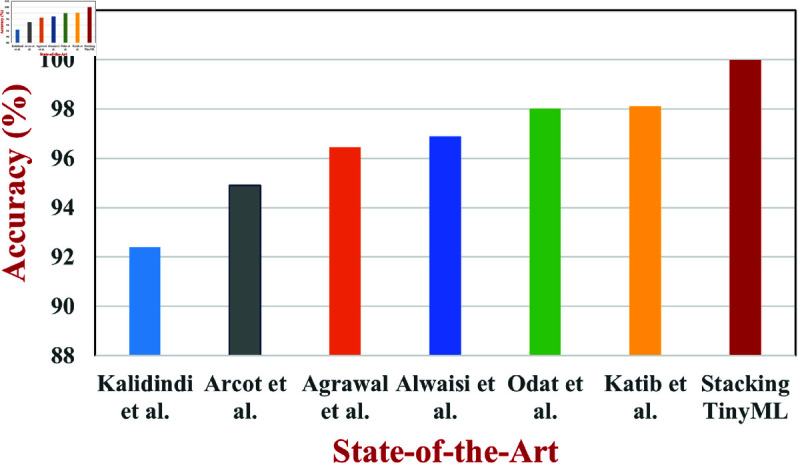
Comparison of accuracy of the proposed stacking TinyML model with the state-of-the-art approaches.

To verify the proposed stacking TinyML model’s capacity for generalisation, it has been also tested using the BoT-IoT dataset. Table 5 shows the comparative analysis of the proposed stacking TinyML model with the different datasets namely the BoT-IoT and ToN-IoT datasets. The model obtained a 98.94% accuracy, with a precision of 98.88%, recall of 98.73%, and F1-score of 98.91%, using the same preprocessing and training pipeline as ToN-IoT. The FPR stayed low at 0.0095, while the specificity is 99.05%. A comparison of several studies on attack detection in IoT networks is shown in Table 6 using ML and TinyML models. Despite the encouraging outcomes of each model, there are still issues with security performance, computational restrictions, and adaptability to attacks. Using CNN-FDSA technique and the BoT-IoT dataset, Kalidindi *et al*. [13] achieved 92.4% accuracy. However, because their strategy depends on pre-established log data and feature selection techniques, it is less flexible when it comes to changing botnet variations. Arcot *et al*. [10] used TinyML to achieve 94.9% accuracy on the USTC-TFC2016 dataset. TinyML-based models were also applied to real-world and Wi-Fi datasets by Agrawal *et al*. [9] and Alwaisi *et al*. [18], who achieved 96.45% and 96.9% accuracy, respectively, but encountered difficulties with security and computation optimization. RF was used by Odat *et al*. [2] on the Malgenome dataset, and while they achieved 98% accuracy, their model had trouble with complicated malware that changes its behaviour on its own. TinyML on ToN-IoT was further enhanced by Katib *et al*. [4], who achieved 98.11% accuracy at the expense of high processing power because of the JOA hyperparameter tuning technique. Fig 4 compared the accuracy of the proposed stacking TinyML model with the other existing approaches.

**Table 5:** Comparison and analysis of proposed model with different datasets.

Dataset	Parameters					
	Accuracy	Precision	Recall	F1-Score	Specificity	FPR
BoT-IoT	0.9894	0.9888	0.9873	0.9891	0.9905	0.0095
ToN-IoT	0.9998	0.9997	0.9991	0.9994	0.9991	0.0009

**Table 6:** Comparison and analysis of performance metrics.

Author/Ref.	Dataset	Technique	Accuracy	Limitation
Kalidindi *et al*. [13]	BoT-IoT	CNN-FDSA	92.4%	Using pre-defined log data and feature selection methods may limit the model’s adaptability to zero-day botnet attacks or unforeseen variants.
Arcot *et al*. [10]	USTC-TFC2016	TinyML	94.90%	Quantization reduces the model’s size and inference time but reduces accuracy.
Agrawal *et al*. [9]	Wi-Fi	TinyAP	96.45%	Microcontroller computational constraints limit the model’s complexity.
Alwaisi *et al*. [18]	Simulation and Real	TinyML	96.9%	Security performance and computational overhead must be optimized.
Odat *et al*. [2]	Malgenome	RF	98%	Complex malware can impersonate innocent apps or dynamically change permissions and API calls, weakening the model against adaptive threats.
Katib *et al*. [4]	ToN-IoT	TinyML	98.11%	The JOA hyperparameter tuning method demands a lot of processing power, making model scaling for huge datasets difficult.
**Proposed Work**	**ToN-IoT**	**Stacking TinyML**	**99.98%**	**The model performs well on the existing dataset, but its efficiency on 6G-IoT networks with millions of devices is uncertain.**

In an IoT environment, where memory, processing power, and energy consumption are limited, traditional ML models including CNN, RF, and other DL architectures are less practical because of their high computational requirements. The large scale of the models and their large inference latency also make them less suitable for real-time attack detection. TinyML leverages DL model optimization techniques in quantization, pruning, knowledge distillation to enable models to work effectively on resource-limited devices with competitive accuracy. Conventional TinyML models lose accuracy as they become more efficient, even with quantization or other optimization. The proposed Stacking TinyML model addresses these problems by aggregating various TinyML classifiers in the stacked ensemble framework. By combining strengths of multiple base models such as DT and NN and a meta-learner named Logistics Regression, stacking improves the precision of prediction relative to using a single TinyML model. The approach can reduce both bias and variance and increases the generality of the approach to various attack.

Balancing the trade-off between accuracy, efficiency, and flexibility, the proposed T-TML stacking model effectively overcomes the limitations addressed in the existing literatures. In contrast with other models with complicated tuning schemes or predefined features, it does not need any feature transformation or selection, and its overall performance is mostly attributed to combining lightweight DT and NN with a LR as the meta classifier. In resource-constrained devices, this structure preserves computationally for computation while ensuring robustness against a wide range of dynamic attack scenarios. Light RF is employed to select features with the purpose of retaining only the most relevant attributes, thereby reducing model complexity and enhancing execution time. The ToN-IoT dataset with diverse and realistic IoT traffic conditions is also utilized to ensure the model is tested against a broad spectrum of threats. By combining ensemble strength, model simplicity, and real-world data training, it is possible to achieve high accuracy (99.98%) with very low power consumption and minimal inference latency, surpassing previous constraints on scalability, efficiency, and flexibility.

### 4.2 Limitation

Even with the proposed model’s great accuracy and efficiency, some limitation of this study still exist. The quality and diversity of the training dataset have a major role in the model’s performance and can impact how well it adapts to new and changing cyberthreats. Furthermore, microcontrollers’ computational limitations restrict the complexity and scalability of the model, which may limit its application in extensive IoT networks as described below.

- Hardware Restrictions: Microcontrollers have limited energy, RAM, and processing capacity, even after the model was optimized for TinyML deployment. This limits the hybrid stacking model’s complexity, which may have an impact on its capacity to detect large-scale, real-time IoT attacks.
- Dataset Dependency: The ToN-IoT dataset, which may not completely cover all potential real-world attack patterns, is used to train and assess the model. Uncertainty surrounds its generalizability to unknown network environments or novel, developing cyberthreats in IoT systems.

## 5 Conclusion and future work

This study uses the ToN-IoT dataset to propose a stacking-based TinyML model for attack detection in IoT networks. Effectively combining lightweight DT and small NN, the stacking model is designed to be deployed on edge devices with limited resources. The proposed model uses stacking-based ensemble learning to improve detection accuracy while consuming 0.01 mW of power and having a low inference latency (0.12 ms). By integrating the advantages of several base models, the stacking technique produces better generalization and robustness against adversarial variations and dramatically increases classification performance when compared to typical TinyML models attaining an accuracy of 99.98% with the rates of precision, recall, F1-score, specificity and FPR at 99.97%, 99.51%, 99.94%, 99.91% and 0.0009 respectively. The model has also been compared with the existing work including Kalidindi *et al*. [13], Arcot *et al*. [10], Agrawal *et al*. [9], Alwaisi *et al*. [14], Odat *et al*. [2] and Katib *et al*. [4] used the different ML and TinyML techniques achieving the accuracies of 92.4%, 94.90%, 96.45%, 96.9%, 98% and 98.11% respectively. Additionally, feature selection using the small RF has contributed to lowering computational overhead while maintaining excellent detection accuracy. The model performs well, however, it has some limitations. Testing on real-world IoT environments is required to assess its resilience against new and changing attack patterns, but its efficacy has been confirmed on the ToN-IoT dataset. Further improvements may be necessary to ensure efficiency for deployment on very diverse and large-scale IoT infrastructures, even though the model is designed for low-power devices.

Future research will concentrate on resolving the issues raised by 6G-IoT networks, such as the high device density, the need for extremely low latency, and the ever-changing security risks. To provide decentralized training among edge devices and lower communication overhead while maintaining data privacy, federated learning will be investigated and addition of explainability tools (like SHAP/LIME) for improved transparency.
